# *Omics* approaches to investigate pre-symbiotic responses of the mycorrhizal fungus *Tulasnella* sp. SV6 to the orchid host *Serapias vomeracea*

**DOI:** 10.1007/s00572-025-01188-6

**Published:** 2025-04-02

**Authors:** Silvia De Rose, Fabiano Sillo, Andrea Ghirardo, Jörg-Peter Schnitzler, Raffaella Balestrini, Silvia Perotto

**Affiliations:** 1 National Research Council, Institute for Sustainable Plant Protection, Strada delle Cacce 73, Torino, I-10135 Italy; 2 Department of Life Sciences and Systems Biology, University of Turin, Viale Mattioli 25, Torino, I-10125 Italy; 3 Research Unit Environmental Simulation (EUS), Helmholtz Zentrum München, Ingolstädter Landstr. 1, D-85764 Neuherberg, Germany; 4 National Research Council, Institute of Biosciences and Bioresources, Via Amendola 165/A, Bari, I-70126 Italy

**Keywords:** Orchid mycorrhiza, *Tulasnella*, *Serapias vomeracea*, Pre-symbiotic events, Interactions

## Abstract

Like other plant-microbe symbioses, the establishment of orchid mycorrhiza (ORM) is likely to require specific communication and metabolic adjustments between the two partners. However, while modulation of plant and fungal metabolism has been investigated in fully established mycorrhizal tissues, the molecular changes occurring during the pre-symbiotic stages of the interaction remain largely unexplored in ORM. In this study, we investigated the pre-symbiotic responses of the ORM fungus *Tulasnella* sp. SV6 to plantlets of the orchid host *Serapias vomeracea* in a dual in vitro cultivation system. The fungal mycelium was harvested prior to physical contact with the orchid roots and the fungal transcriptome and metabolome were analyzed using RNA-seq and untargeted metabolomics approaches. The results revealed distinct transcriptomic and metabolomic remodelling of the ORM fungus in the presence of orchid plantlets, as compared to the free-living condition. The ORM fungus responds to the presence of the host plant with a significant up-regulation of genes associated with protein synthesis, amino acid and lipid biosynthesis, indicating increased metabolic activity. Metabolomic analysis supported the RNA-seq data, showing increased levels of amino acids and phospholipids, suggesting a remodelling of cell structure and signalling during the pre-symbiotic interaction. In addition, we identified an increase of transcripts of a small secreted protein that may play a role in early symbiotic signalling. Taken together, our results suggest that *Tulasnella* sp. SV6 may perceive information from orchid roots, leading to a readjustment of its transcriptomic and metabolomic profiles.

**Supplementary Information:**

The online version contains supplementary material available at 10.1007/s00572-025-01188-6.

## Introduction

Symbioses between plants and microbes play a crucial role in the functioning of ecosystems, enhancing plant growth, health and productivity. The establishment of symbiotic relationships requires a communication system that enables plants and microbes to signal their reciprocal presence. Upon recognition of available symbiotic partners, metabolic pathways need to be activated to facilitate physical contact and establishment of the symbiosis. Among plant-microbe symbioses, mycorrhiza stands out as a particularly complex form of association that involves the roots of most terrestrial plant species and many soil fungi (Smith and Read 2010; Genre et al. 2020; Shi et al. 2023).

Pre-symbiotic events that occur before physical contact between mycorrhizal fungi and their host plant have been investigated mainly in arbuscular mycorrhiza (AM), the most ancient symbiosis in terrestrial plants (Genre et al. 2020). Here, plant and fungal chemical compounds involved in recognition are released in the rhizosphere. In particular, plants capable of forming AM release small molecules called strigolactones (SLs), phytohormones that are members of the large group of sesquiterpene lactones, initially discovered as inducers of seed germination in parasitic plants such as *Striga* and *Orobanche* (Matusova et al. 2005). In the pre-symbiotic stages of the AM symbiosis, SLs can induce extensive hyphal branching in the germinating spores of AM fungi (Buée et al. 2000). Before branching, activation of AM fungal genes related to mitochondrial activity and lipid catabolism have been observed, as well as increased fungal respiration rate and mitochondrial density, suggesting a metabolic switch in the AM fungus upon perception of the plant SLs (Besserer et al. 2006, 2008; Lanfranco et al. 2018). In response to the host plant, AM fungi release two types of related signalling molecules, namely lipo-chito-oligosaccharides (LCOs, Maillet et al. 2011), and chito-oligosaccharides (COs, Genre et al. 2013). LCOs were first discovered as being released by symbiotic nitrogen-fixing rhizobia in the pre-symbiotic stages of the legume nodule symbiosis (Dénarié et al. 1996; Murray et al., 2011). The structurally similar LCOs produced by rhizobia and by AM fungi stimulate the host plant responses through the Common Symbiotic Signalling Pathway (CSSP), a shared signal transduction pathway (Kistner et al. 2005; Gutjahr and Parniske 2013; Genre and Russo 2016). Intriguingly, core genes of the CSSP were revealed in the genome of plants forming different types of endosymbioses, i.e., associations where the symbiotic microbe is hosted inside the plant cells (Delaux et al. 2014; Radhakrishnan et al. 2020). This genome comparison included orchids, a large family of flowering plants forming typical endomycorrhizal associations with fungi mainly belonging to the Basidiomycetes (Smith and Read 2010). Expression of *Bletilla striata* and *Dendrobium nobile* homologs of AM-related calcium and calmodulin-dependent protein kinase (CCaMK), a core gene of the CSSP, was activated during orchid mycorrhizal (ORM) symbiosis (Miura et al. 2018; Xing et al. 2020). These findings would indicate that early events in ORM may include chemical signals able to induce a response in the symbiotic partners.

Despite the genetic and morphological similarities between AM and ORM (Perotto and Balestrini 2024), the fungi involved in these two symbioses are taxonomically very different. Most fungi found in ORM are Basidiomycetes (Dearnaley et al. 2012), and many fungal species associated with ORM are also capable of forming ectomycorrhiza (ECM) on other host plants (Yagame et al. 2011). Early responses of the ECM fungus *Hebeloma cylindrosporum* to symbiosis have highlighted early transcriptional responses to host signals, involving mycorrhiza-induced small secreted proteins (MiSSPs), such as in AM symbiosis, and a reduced set of CAZymes that likely modify plant responses to facilitate symbiosis establishment (Pellegrin et al. 2015; Doré et al. 2017). On the other hand, the early stages of interaction between the ECM fungus *Pisolithus microcarpus* and *Eucalyptus grandis* involved significant changes in metabolite secretion occurring within the first hour of contact, with secretion of compounds such as phenylpropanoids and fatty acids (Plett et al. 2021).

Although *-omics* approaches have been employed to understand the complexity of the ORM interactions over the last years, the pre-symbiotic stages of plant-fungus interactions have been poorly investigated, and most studies have focused on the modulation of plant and fungal metabolism in established mycorrhiza (Perotto et al. 2014; Zhao et al. 2014; Fochi et al. 2017a, b; Miura et al. 2018; Ghirardo et al. 2020; Valadares et al. 2021; De Rose et al. 2023a; Rose et al. 2023b).

In the natural environment, the germination of orchid seeds and the early stages of plant development typically require colonization by a fungal symbiont. This is due to the fact that the minute dust-like orchid seeds contain minimal or no stored energy reserves, which are provided by the ORM fungus (Arditti 1992; Smith and Read 2010). Nevertheless, asymbiotic seed germination and orchid growth can be achieved on artificial media that provide the plant with all the nutrients they require, including simple carbon sources (Knudson 1922). In order to investigate pre-symbiotic events in ORM, we developed a dual in vitro cultivation system where the fungal isolate *Tulasnella* sp. SV6 was cultivated in the presence of asymbiotic plantlets of the terrestrial orchid host *Serapias vomeracea*. The genus *Tulasnella* (Tulasnellaceae, Agaricomycotina, Basidiomycetes) is among the most prevalent ORM fungi in both temperate and tropical climates (Dearnaley et al. 2012). In particular, this study focused on the pre-symbiotic responses of *Tulasnella* sp. SV6 to the presence of the orchid host. To this end, the fungal mycelium was collected before physical contact with the plantlet, and the fungal transcriptome and metabolome were studied to reveal possible remodeling of the fungal metabolism at this stage of the interaction.

## Materials and methods

### Biological material

Seeds of *S. vomeracea* (BURM.) BRIQ. were collected during summer 2021 from mature capsules of wild plants grown in the locality of Cairo Montenotte (SV, Italy; Latitude 44.406944 N, Longitude 8.319795 E) and stored at 4 °C. The fungal isolate SV6 (MUT4178), belonging to the *Tulasnella* genus, was originally isolated from *Serapias vomeracea* roots grown in Northern Italy (Girlanda et al. 2011) and stored in the Mycotheca Universitatis Taurinensis (MUT) at the University of Turin. The fungal isolate was grown and sub-cultured on solid oatmeal agar (OA, 0.3%) at 25 °C in the dark. To assess fungal growth rate on the M551 medium, used in the co-inoculation experiment, the mycelium was transferred to Petri dishes containing solid M551 growth substrate and it was used as inoculum. A similar growth rate was observed for *Tulasnella sp. SV6* on OA and M551 media (Fig. S1).

### Asymbiotic germination of *S. vomeracea*

To obtain asymbiotic plantlets of *S. vomeracea*, seeds were surface sterilized with a solution of 1% (v/v) sodium hypochlorite and 0.1% (v/v) Tween-20 for 20 min, followed by three 5-min rinses with sterile distilled water, re-suspended in sterile water and placed on 9 cm Petri dishes containing solid BM1 culture medium (Van Waes and Deberg, 1986). Plates were incubated at 20 °C in darkness. After 90 days, protocorms reached 3–4 mm in diameter and were exposed to natural light for an additional 30 days at room temperature. Once a leaf primordium formed at the top of the protocorms, they were transferred to 50 mL centrifuge tubes containing Malmgren Modified Terrestrial Orchid Medium (M551, PhytoTechnology Laboratories^®^) with sucrose 10% (w/v) at pH 5.8, prepared following the manufacturer’s instructions, and grown at room temperature and natural light until seedling development. Once the seedlings showed developed leaf blades and rootlets, they were transferred to sterilized magenta jars containing fresh M551 medium. The procedure is shown in Figure S2. Just before the addition of the fungal mycelium, autoclaved shape-adapted cellulose membranes were positioned on top of the solid medium in the magenta jars, with a central hole for the orchid plantlet.

### Experimental design and fungal sample preparation for transcriptomic and metabolomic analyses

To investigate the influence of *S. vomeracea* plantlets on the fungal transcriptome and metabolome, four plugs of *Tulasnella* sp. SV6 mycelium actively growing on M551 medium were placed on the autoclaved cellulose membranes, at the four corners of the magenta jars containing the asymbiotic orchid plantlet (Figure S3, a). The *Tulasnella* sp. SV6 mycelium was collected 5 days post inoculum (dpi), before the mycelium encountered the roots (PRESYMB sample, Figure S3, b). A plug of *Tulasnella *sp. SV6 mycelium actively growing on M551 medium was also inoculated on autoclaved cellulose membranes placed on top of M551 medium in Petri plates, to yield free-living mycelium (FLM sample) as asymbiotic control. Three biological replicates for each condition (PRESYMB and FLM) were prepared for transcriptomics, and four biological replicates for metabolomics. After sampling, all samples were frozen in liquid nitrogen and stored at -80 °C.

### Transcriptomic analysis

For the RNA-seq experiment, fungal mycelia for each tested condition (PRESYMB, FLM) were freeze-dried and finely homogenized with a TissueLyser (25 Hz, 1 min, twice, Qiagen Diagnostic GmbH, Hilden, Germany). RNA was extracted from ca. 80 mg of biological material from each biological replicate using the “pine tree” CTAB-based method (Chang et al. 1993). The RNA was then eluted in RNA-seq free water and quantified with NanoDrop 2000 and Qubit 4 Fluorometer (Thermo Fisher Scientific, Waltham, MA, USA). IGA Technology Services (Udine, Italy) carried out the library preparation and RNA sequencing. In detail, Universal Plus mRNA-Seq kit (Tecan Genomics, Redwood City, CA) was used for library preparation, following the manufacturer’s instructions (library type: fr-secondstrand). RNA samples were quantified and quality tested with an Agilent 2100 Bioanalyzer RNA assay (Agilent technologies, Santa Clara, CA) or by Caliper LabChip GX (PerkinElmer, Waltham, MA). Libraries were then sequenced on paired-end 150 bp mode on NovaSeq 6000 (Illumina, San Diego, CA).

Reads were aligned to the *Tulasnella calospora* AL13/4D v1.0 genome available at https://mycocosm.jgi.doe.gov/Tulca1/ (Kohler et al. 2015) by using STAR v2.7.10a (Spliced Transcripts Alignment to a Reference; Dobin et al. 2013). To convert SAM data into BAM files and index them, the Samtools 1.11v was used (Li et al. 2009). Reads aligned on the reference genome were counted using HTseq-count 2.0.2v (Anders et al., 2014), and exon junctions and gene overlaps were mapped using the intersection method.

To identify differentially expressed genes (DEGs), raw read counts were imported into the DESeq2 tool version 1.34.0 (Love et al. 2014). Following default DESeq2 normalization of the count data (median of ratios method) and correction for multiple testing, DEG identification was carried out through Wald test using FLM samples as controls. The three biological replicates per condition were employed to compute read count variation. An adjusted p-value threshold of 0.05 was employed to identify DEGs, that were functionally annotated in silico using Blast2GO v5.2.5 (Conesa et al. 2005), with their related Gene Ontology (GO) terms subsequently assigned. To perform a GO enrichment analysis and to provide a summary of the functions and pathways associated with the obtained sets of DEGs, Blast2GO v5.2.5 was employed. Raw reads were submitted to the NCBI Sequence Read Archive (SRA) under BioProject accession number PRJNA1194787.

### Metabolomic analysis

A total of 8 frozen samples (four biological replicates for each condition; PRESYMB and FLM) were freeze-dried at -50 °C under vacuum (Alpha 1–4 LDplus, Christ, Osterrode, Germany) and finely ground with TissueLyser (25 Hz, 1 min, Qiagen Diagnostic, Hilden, Germany). Approximately 10 mg of dried fine powder per sample was extracted following the protocol used by Bertić et al. (2021). Briefly, 800 µL of methanol/2-propanol/water solution (1/1/1, v/v/v) containing 50 µL of an internal standard (IS) mixture were added to the sample (Table S1). The mixture was vortexed for 1 min, sonicated for 10 min at 5 °C in an ultrasonic bath and then centrifuged at 9300 *g* for 10 min at 5 °C. The supernatant (400 µL) was recovered and the centrifugation process was repeated. The remaining 400 µL of supernatant was collected to obtain a total of 800 µL of the extracted metabolite solution. The supernatant was dried by SpeedVac (Univapo 150 H, Uniequip, Planegg, Germany) and re-dissolved in 350 µL of 50% (v/v) acetonitrile in water. The solution was mixed and centrifuged at 9300 *g* for 10 min at 5 °C, and 300 µL of supernatant was collected and transferred in amber glass vials for metabolomics analysis.

Non-targeted metabolomic analysis was performed by ultra-performance liquid chromatography (UPLC) ultra-high resolution (UHR) tandem quadrupole/time-of-flight (QqToF) mass spectrometry (MS), as described in Bertić et al. (2021). The system consists of an Ultimate 3000RS UPLC (Thermo Fisher Scientific, Bremen, Germany), a Bruker Impact II (QqToF) and an Apollo II electrospray ionization source (Bruker Daltonics, Bremen, Germany). Metabolites were separated by reversed-phase liquid chromatography (RPLC) and by hydrophilic interaction liquid chromatography (HILIC), each run separately and eluted using H_2_O + 0.1% (v/v) formic acid (solvent A) and acetonitrile + 0.1% (v/v) formic acid (solvent B) (see Bertić et al. 2021 for details). The spectra were acquired in both positive (+) and negative (−) ionization modes. Data were processed as outlined in Bertić et al. (2021), using the Metaboscape v4.0 software (Bruker Daltonics) and the parameters listed in Table S2.

Results of RP and HILIC analyses were manually merged and zero values were replaced with random numbers below the threshold value of 700 area unit. Data were normalized for the dry mycelium weight. Compounds were annotated as described in Bertić et al. (2021), matching MS/MS spectra using the libraries ‘All Spectra’ in MoNA (Mass Bank of North America, https://mona.fiehnlab.ucdavis.edu), MS-DIAL Lipids (http://prime.psc.riken.jp), MassBank (https://massbank.eu/MassBank/), Vaniya/Fiehn Natural Products Library (available through MoNA) and GNPS (https://gnps.ucsd.edu). Principal component analysis (PCA) was calculated using the normalized peak areas, logarithmically transformed and pareto scaled in MetaboAnalyst 6.0. Log_2_FC were calculated using FLM as control, applying Student t-test and Benjamini–Hochberg correction to discriminate significant values (adj. p-value score < 0.05).

## Results

### Transcriptomic profile of *Tulasnella *sp. SV6 during pre-symbiotic growth in the presence of *S. vomeracea* plantlets

**Fig. 1:**
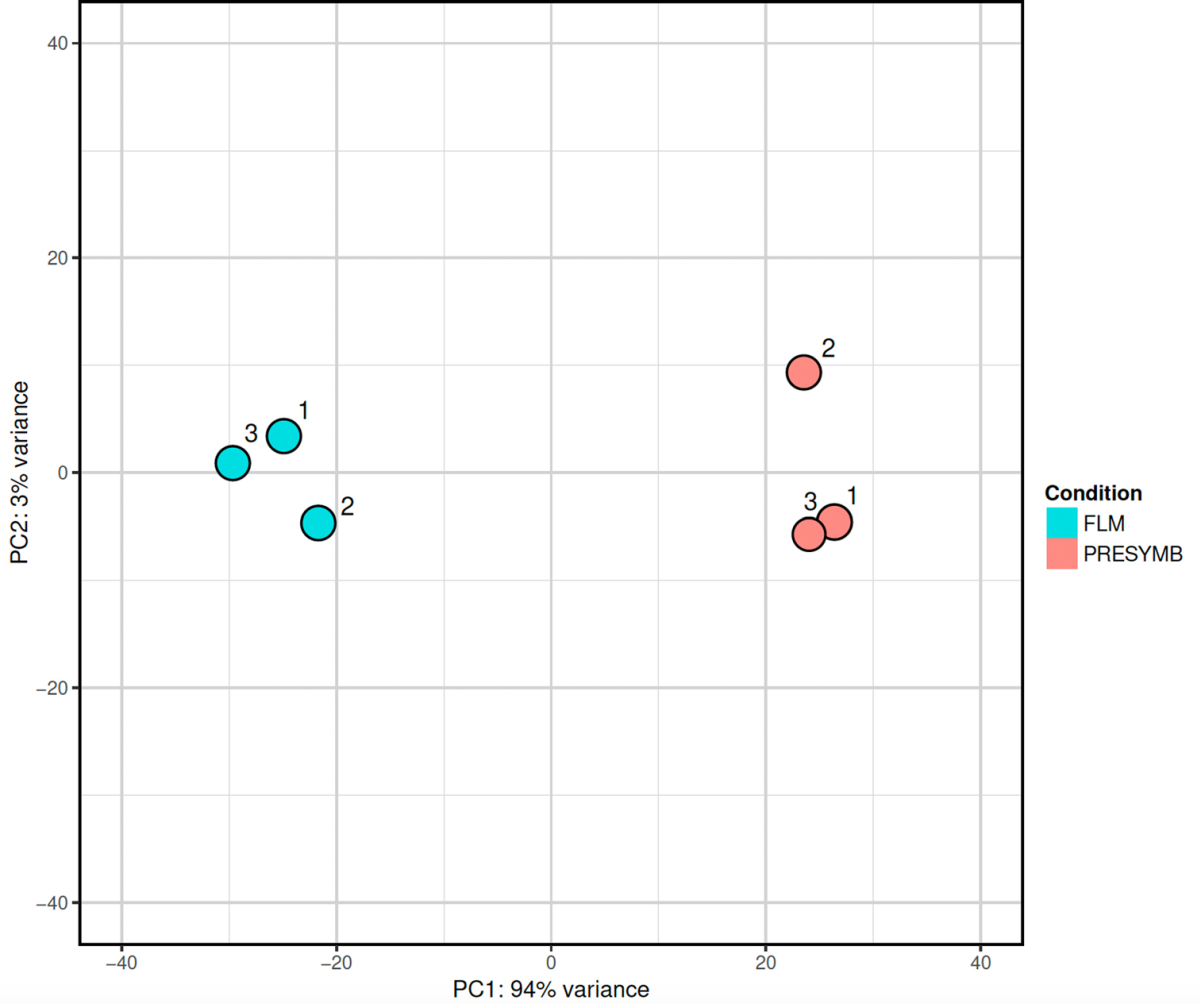
Principal components analysis (PCA) of normalized read counts of all samples used in RNAseq experiment

Sequencing of RNA samples produced an average of 27,433,879 raw reads per sample (ranging from 24,260,989 to 32,378,146; Table S3). Overall, reads mapping on the *Tulasnella calospora* AL13/4D v1.0 reference genome resulted in an average mapping rate of 85.08%. The PCA of normalized read count data (Fig. 1) showed that RNA samples grouped into two distinct groups, the FLM and the PRESYMB samples being well separated by the first principal component (PC1; 94% of variance).

**Fig. 2:**
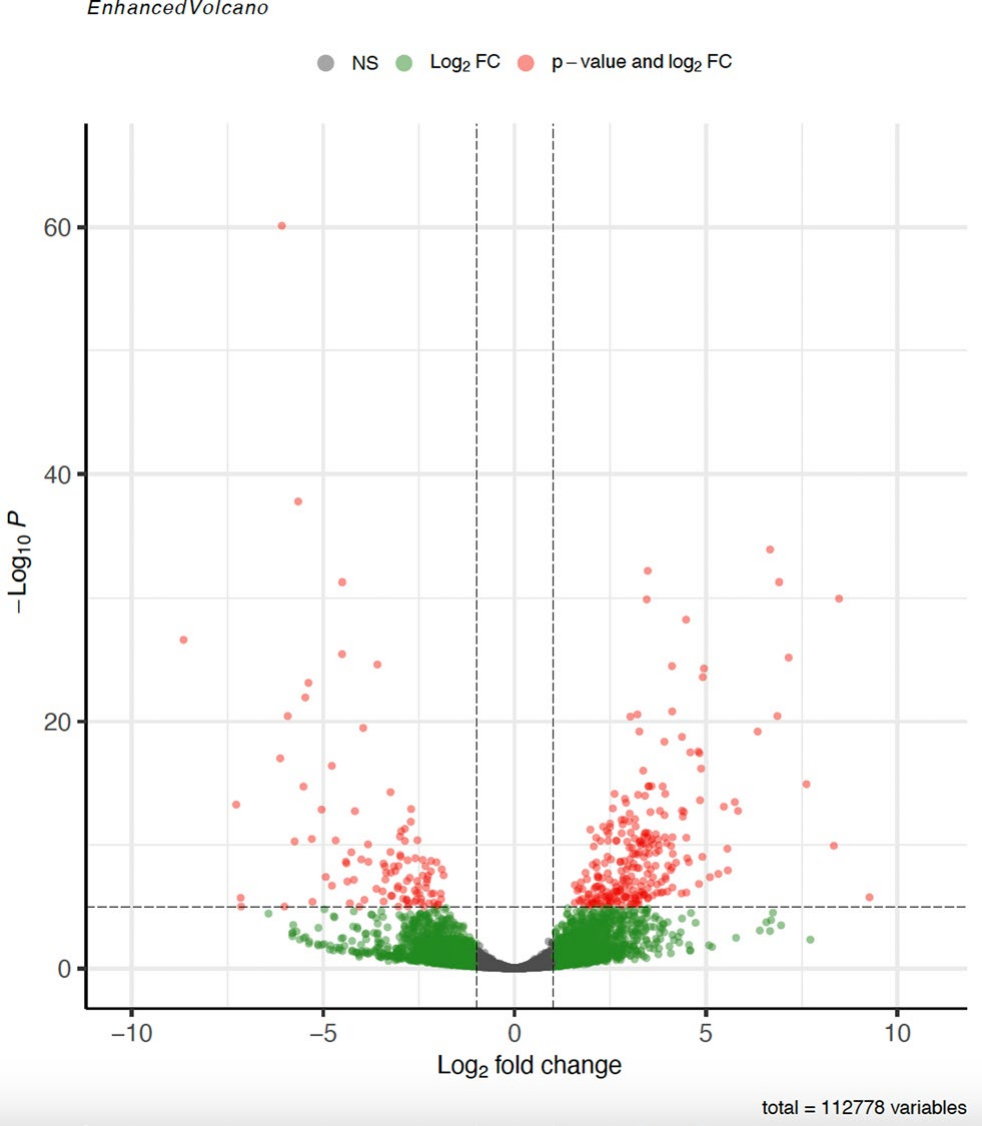
Volcano plots showing identified DEGs in the PRESYMB sample as compared with the FLM sample (control condition). Significant up-and down- regulated genes were represented by red dots (Log_2_FC > 1 or < -1, p-adjusted value < 0.05). Green dots represent genes with a Log_2_FC > 1 or < -1 but a not-significant p-adjusted value (p adjusted value > 0.05). Grey dots represented genes showing a Log_2_FC ranging from − 1 and 1 and a not-significant p-adjusted value (p-adjusted value > 0.05)

**Fig. 3:**
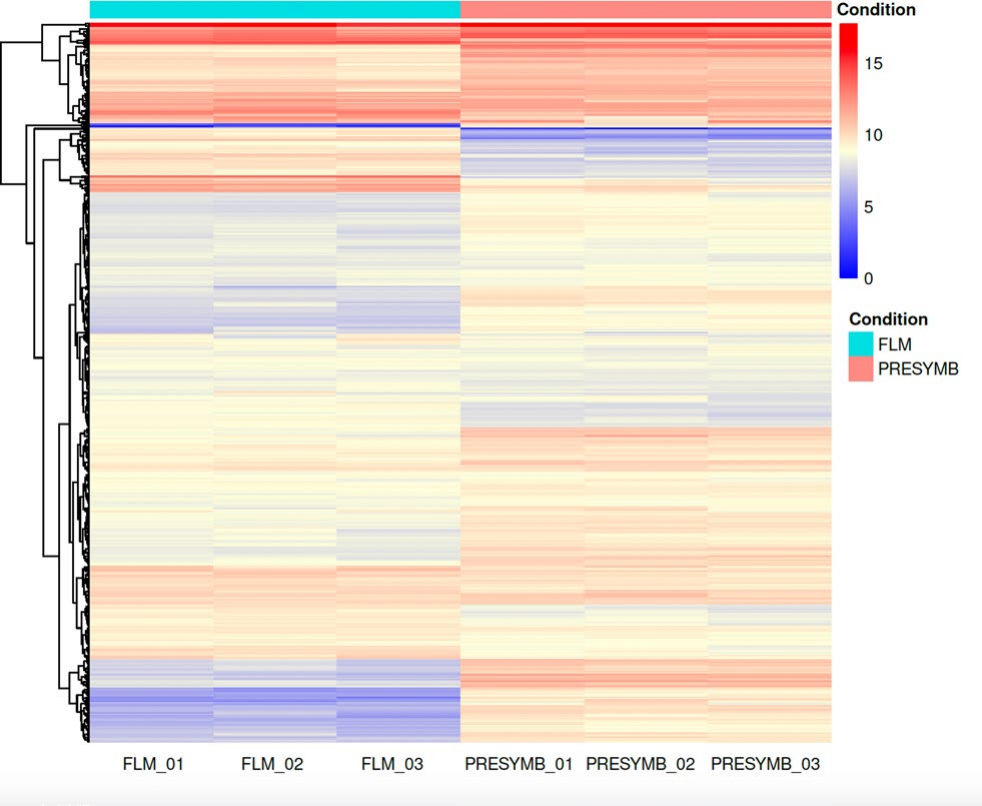
Heatmap and hierarchical clustering of the 500 genes showing the most divergent expression in the three biological replicates of PRESYMB and FLM, using the McQuitty algorithm. The heatmap shows the gene expression patterns in the two conditions. Different color intensity indicates different levels of expression based on normalized read count

Gene expression in the *Tulasnella* sp. SV6 mycelium during pre-symbiotic (PRESYMB) and free-living (FLM) growth was compared to identify differentially expressed genes (DEGs) in the presence and in the absence of the orchid plantlet. A total of 1,669 genes (out of 19,659 *T. calospora* Al13/4D v.1 total genes) were found to be differentially regulated with adj. *p*-value < 0.05. In Fig. 2, a Volcano plot shows the distribution of DEGs detected in PRESYMB. When compared to FLM, transcriptomic reprogramming during pre-symbiotic growth involved 1002 up-regulated and 667 down-regulated genes, respectively. The heatmap and hierarchical clustering in Fig. 3 depicts the significant relative changes in the expression of the top 500 genes showing the most divergent expression (gene expression patterns based on transcript count) between PRESYMB and FLM samples.

The complete list of up-regulated and down-regulated DEGs in the PRESYMB sample, as compared to the FLM sample, can be found in Table S4, and the top 20 up-regulated and down-regulated genes with a known putative function are listed in Tables 1 and 2, respectively. The top 20 up-regulated DEGs all showed a Log_2_FC > 5, the most up-regulated one coding for a putative glutathione S-transferase (transcript id: 31195, Log_2_FC 9.27). A DEG coding for a small secreted protein was also strongly up-regulated (transcript id: 246100, Log_2_FC 7.15). Among these top 20 up-regulated DEGs were also two putative aldo/keto reductases (transcript ids: 28543, Log_2_FC 7.62 and 4711, Log_2_FC 6.67), a tryptophan synthetase (transcript id: 185677, Log_2_FC: 6.74), two putative homo-isocitrate dehydrogenase LYS12, involved in lysine biosynthesis (transcript id: 165648, Log_2_FC: 5.15), a putative metallo-endopeptidase belonging to the M35 family (transcript id: 178320, Log_2_FC: 6.67) and a tetratricopeptide repeat (TPR)-like protein (transcript id: 78871, Log_2_FC: 6.70).

**Table 1:** List of top 20 up-regulated DEGs in the PRESYMB samples

Transcript id	Log_2_FC	Log_2_FC SE	Adj. *p*-value	Description
31195	9.27	1.70	1.75E-06	glutathione S-transferase family protein
230917	7.73	2.18	4.74E-03	S1/P1 nuclease-domain-containing protein
28543	7.62	0.88	1.21E-15	Aldo/keto reductase
246100	7.16	0.64	6.76E-26	small secreted protein
104862	6.96	1.62	3.29E-04	flavo protein WrbA
21363	6.87	0.68	3.68E-21	reverse transcriptase domain-containing protein
185677	6.75	1.38	3.07E-05	tryptophan synthetase
78871	6.70	1.47	1.24E-04	TPR-like protein
4711	6.67	0.52	1.31E-34	putative aldo-keto reductase
178320	6.67	1.66	9.44E-04	M35 family metallo-endopeptidase
21793	6.58	1.48	1.84E-04	DJ-1/PfpI family protein
32761	6.35	0.65	6.60E-20	minor allergen Alt a 7
106210	5.84	0.73	1.76E-13	60 S ribosomal protein L15
20463	5.78	1.58	3.35E-03	hypothetical protein SCP_0703600
17873	5.75	0.70	3.44E-14	FAD/NAD(P)-binding domain-containing protein
243502	5.56	0.79	2.04E-10	hypothetical protein CB0940_10658
5838	5.32	0.85	2.22E-08	probable nucleolar protein 5 − 2
165648	5.16	1.67	1.82E-02	putative LYS12 - Homo-isocitrate dehydrogenase
8665	5.10	0.83	4.13E-08	protein FAR1-RELATED SEQUENCE 5-like
9090	5.08	1.59	1.34E-02	GPN-loop GTPase QQT2 isoform X2

**Table 2:** List of top 20 down-regulated DEGs in the PRESYMB samples

Transcript id	Log_2_FC	Log_2_FC SE	Adj. *p*-value	Description
18717	-8.64	0.75	2.47E-27	putative GPI-anchored cupredoxin
18719	-7.27	0.89	5.48E-14	putative extracellular serine-rich protein
12253	-7.15	1.31	1.95E-06	Rhamnogalacturonase B
17088	-7.14	1.40	9.77E-06	cytochrome P450
30377	-6.12	0.66	9.79E-18	hypothetical protein COCCADRAFT_36097
26982	-5.80	1.55	2.51E-03	WD40 repeat-like protein
29516	-5.79	1.47	1.20E-03	recombinase. putative
244650	-5.78	1.34	3.07E-04	2-oxoglutarate metabolism-related protein. putative
27833	-5.74	0.79	5.26E-11	putative GPI-anchored cupredoxin
30761	-5.70	1.43	1.04E-03	S1/P1 nuclease-domain-containing protein
20821	-5.65	0.41	1.68E-38	Hsp20/alpha crystallin family protein
4369	-5.51	0.64	1.89E-15	tyrosinase
241343	-5.38	0.50	7.51E-24	Di-copper centre-containing
92348	-5.28	1.00	4.12E-06	serine/arginine repetitive matrix protein 1 isoform X3
20659	-5.14	1.57	1.10E-02	Bet v1-like protein
241500	-5.04	0.62	1.38E-13	uncharacterized protein BJ212DRAFT_1482028
32580	-4.97	1.51	1.08E-02	Endoglucanase gh5-1
34786	-4.93	0.80	3.97E-08	cytochrome P450
17292	-4.77	0.53	3.91E-17	UMTA protein
25833	-4.73	1.67	3.53E-02	pectin lyase fold/virulence factor

In addition to the tryptophan synthetase and the LYS12 genes, additional genes involved in the biosynthesis of amino acids were induced in the PRESYMB samples (Table S4), such as arginine (transcript id: 228403, Log_2_FC: 3.16 and transcript id: 224256, Log_2_FC: 2.16), asparagine (transcript id: 182504, Log_2_FC: 2.38), histidine (transcript id: 233647, Log_2_FC: 1.60), leucine (transcript id: 96719, Log_2_FC: 2.01 and transcript id: 142459, Log_2_FC: 2.59), and lysine biosynthesis (transcript id: 109796, Log_2_FC: 3.20 and transcript id: 240710, Log_2_FC: 4.13). The shikimate pathway, leading to the synthesis of phenylalanine, tyrosine and tryptophan, was also activated in the PRESYMB sample (transcript id: 172333, Log_2_FC: 4.11). Some genes coding for glycosyl hydrolases (GH) were identified as being up-regulated. Three of them belong to the GH5 family (transcript ids: 93410, 188339 and 240794, Log_2_FC 1.88, 1.76 and 1.75, respectively), i.e., endoglucanases reported to be involved in plant-cell remodeling during early fungal colonization (Zhang et al. 2018), one to the GH32 family (transcript id: 23305, Log_2_FC 3.97), which includes invertases, and one to the GH79 family (transcript id: 162910, Log_2_FC 1.14).

The top 20 down-regulated DEGs with known putative functions in the PRESYMB samples are listed in Table 2. Among them, two genes coded for putative GPI-anchored cupredoxin (transcript id: 18717, Log_2_FC: -8.64 and transcript id: 27833, Log_2_FC: -5.74), the first being the most down-regulated gene in the PRESYMB samples. Two genes coding for cytochrome P450s were also among the top 20 down-regulated genes (transcript id: 17088, Log_2_FC: -7.14 and transcript id: 34786, Log_2_FC: -4.93, respectively), as well as some CAZYmes, namely a rhamnogalacturonase B (transcript id: 12253, Log_2_FC: -7.15), an endoglucanase GH5-1 (transcript id: 32580, Log_2_FC: -4.97) and a pectin lyase (transcript id: 25833, Log_2_FC: -4.73).

**Fig. 4:**
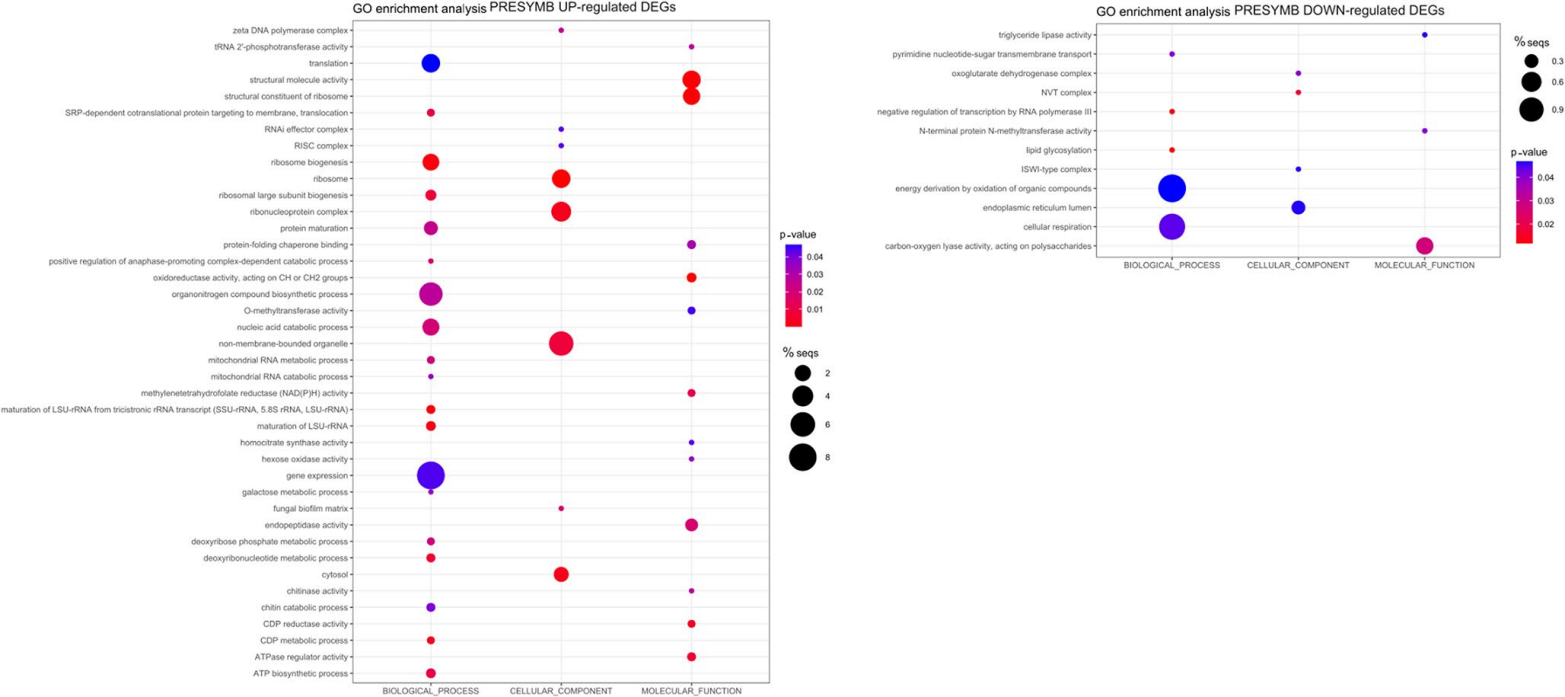
Bubble plots showing GO-enriched terms classified as Biological Process (BP), Molecular Function (MF) and Cellular Component (CC) in detected up and down-regulated DEGs. The x-axis shows the three functional GO categories, and the y-axis reports the associated GO terms. Sizes of bubbles are proportional to the percentage of genes in the selected DEG list belonging to a pathway divided by the corresponding percentage in all reference gene list, while bubble color indicates the significance of the enriched term (False Discovery Rate values) as calculated by the enrichment analysis by Blast2GO

A GO enrichment analysis was conducted to identify the biological processes, cellular compartments and molecular functions modulated in the ORM fungus during pre-symbiotic interactions with the orchid plantlet (Fig. 4). The GO terms enriched in the PRESYMB up-regulated transcriptome for biological processes were mainly related to ribosome biogenesis, organonitrogen compound biosynthetic process; the GO terms enriched for cellular components were nonmembrane-bound organelles, cytosol and ribosomes; the GO terms enriched for molecular functions were again related to ribosome and structural molecule activity (Fig. 4).

### Metabolomic profiles of *Tulasnella *sp. SV6 during pre-symbiotic growth with *S. vomeracea* plantlets

**Fig. 5:**
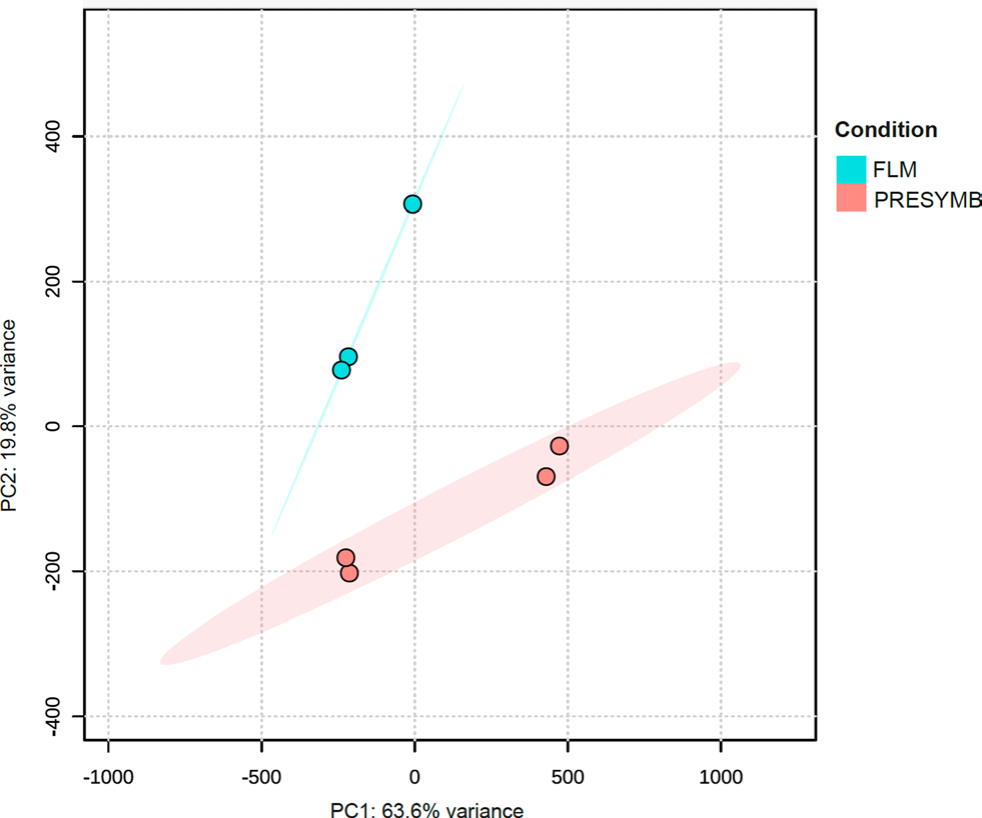
Principal components analysis (PCA) of metabolite-related mass features (m.f.) measurements in all samples

The metabolomic profile of PRESYMB samples was compared with the profile of FLM samples by PCA (Fig. 5). The first two PCA components (PC1 and PC2) explained 83.4% of the total metabolome variance and showed separation between PRESYMB and FLM samples.

**Fig. 6:**
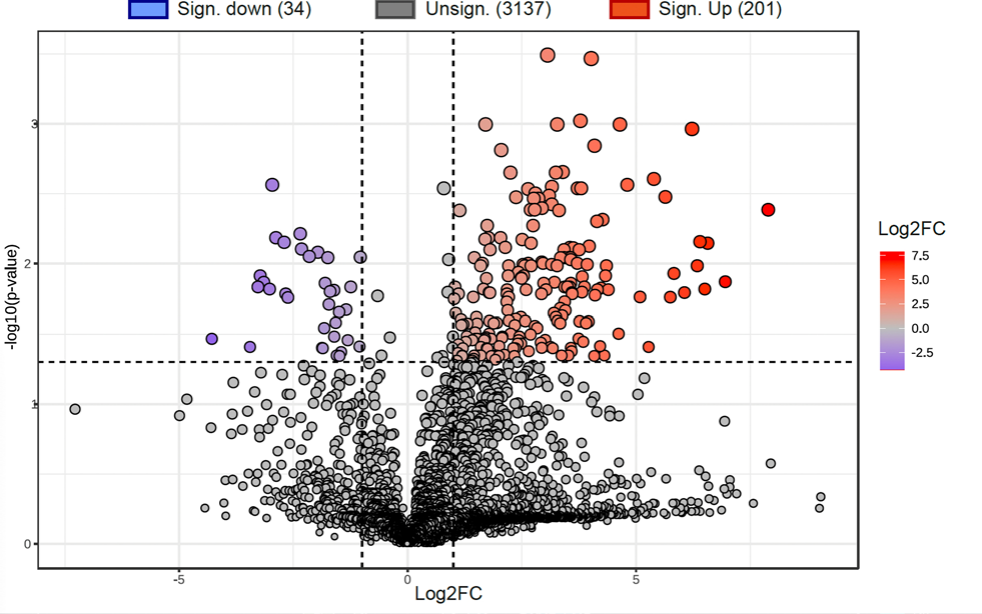
Volcano plots showing identified m.f. in PRESYMB as compared to FLM (control condition). Significant up- and down- accumulated m.f. were represented by red dots (Log_2_FC > 1, p-adjusted value < 0.05) and blue dots (Log_2_FC < -1, p-adjusted value < 0.05), respectively. Grey dots represented m.f. showing a not-significant p-adjusted value (p-adj > 0.05)

A total of 3372 mass features could be detected among the two different conditions. Significantly different abundances of mass features (235 with an adj. p-value < 0.05) were detected in the PRESYMB samples with respect to FLM samples. The results are shown in the volcano plot in Fig. 6, and the complete list of Differentially Accumulated Mass Features (DAMs) is provided in Table S5. Among them, 36 compounds were putatively annotated and are listed in Table 3. Most of them (30 out of 36 DAMs) were more abundant in the PRESYMB samples than in the FLM samples and include several lipids, mostly phospholipids such as phosphatidylcholines (PC) and lysophosphatidylcholines (LPC). Among the phospholipids accumulated in the PRESYMB samples were PC(18:3_20:5), PC(18:2_18:3), PC(16:0/0:0), PC(35:4), PC(18:1), PC(18:2), PC(9:0), LPC(14:0-SN1), LPC(18:3-SN1) and LPC(17:2-SN1). By contrast, a lower abundance in the PRESYMB samples, as compared to FLM, was observed for LPC(18:1) and phosphatidylethanolamine PE(O-15:1_3:0). Other lipids and lipid-like molecules more abundant in the PRESYMB than in the FLM samples were linolenic acid (Log_2_FC: 3.57) and compounds annotated as valeryl-carnitine (Log_2_FC of 2.64 and 1.83) or azelaic acid (Log_2_FC:1.77). Other metabolites more abundant in the PRESYMB samples correspond to amino acids such as proline, pipecolinic acid, phenylalanine (Log_2_FC of 6.22, 4.21 and 3.72, respectively), the dipeptides isoleucylvaline (Log_2_FC: 3.18) and leucylproline (Log_2_FC: 3.62), as well as secondary metabolites like cinnamic acid (Log_2_FC: 3.80) and two sesquiterpenoids like muurolladie-3-one and aspergillusene A (Log_2_FC: 5.75 and 1.52 respectively). Among the metabolites that were less abundant in PRESYMB than in FLM samples, trehalose-6-phosphate showed the most negative Log_2_FC (-4.28).

**Table 3:** List of annotated mass features detected with higher or lower abundance in the PRESYMB condition, as compared to FLM

Id	RT [min]	m/z meas.	Name	Molecular Formula	Log_2_FC	*P*-adj. value
rp3812	25.91	802.5343	PC 18:3_20:5	C46H76NO8P	6.95	0.013455
rp3813	25.92	780.5524	PC 18:2_18:3	C44H78NO8P	6.50	0.015172
rp1009	12.34	116.0704	PROLINE	C5H9NO2	6.22	0.001088
rp1603	17.61	219.1745	MUUROLLADIE-3-ONE	C15H22O	5.74	0.017328
rp2551	19.16	496.339	PC (16:0/0:0)	C24H50NO7P	5.38	0.002478
rp3828	26.49	768.5526	PC 35:4	C43H78NO8P	5.27	0.039068
rp311	6.82	298.0964	5-S METHYLTHIOADENOSINE; LC-TDDA; CE30	C11H15N5O3S	4.64	0.00101
h177	11.68	130.085	PIPECOLINIC ACID	C6H11NO2	4.21	0.038573
rp2871	20.41	524.3702	1-STEAROYL-SN-GLYCERO-3-PHOSPHOCHOLINE	C26H54NO7P	4.09	0.001435
rp83	4.77	149.0592	CINNAMIC ACID	C9H8O2	3.79	0.002891
rp82	4.77	166.086	PHENYLALANINE	C9H11NO2	3.72	0.002891
h168	5.25	534.2946	PC (16:0/0:0)	C24H50NO7P	3.59	0.016078
h19	6.16	104.0699	4-AMINOBUTANOATE	C4H9NO2	3.57	0.03964
rp2923	20.69	279.2312	LINOLENIC ACID	C18H30O2	3.56	0.009063
rp2286	18.44	518.3237	LPC 18:3-SN1	C26H48NO7P	3.51	0.044945
rp1821	17.86	536.3341	PC 18:1	C26H50NO8P	3.34	0.020771
rp213	6.2	231.1703	ISOLEUCYLVALINE	C11H22N2O3	3.17	0.039899
rp1875	17.93	534.3195	PC 18:2	C26H48NO8P	3.15	0.002815
rp3119	21.86	282.2786	LAUROCAPRAM	C18H35NO	2.93	0.009728
rp540	8.24	246.1699	VALERYL-CARNITINE; AIF; CE30; CORRDEC	C12H23NO4	2.63	0.009931
rp2318	18.48	506.3224	LPC 17:2-SN1	C25H48NO7P	2.55	0.010345
rp2462	18.84	267.1718	TRI-ISOBUTYLPHOSPHATE	C12H27O4P	2.17	0.018705
rp568	8.57	246.1699	VALERYL-CARNITINE; AIF; CE30; CORRDEC	C12H23NO4	1.82	0.034181
rp1138	13.9	211.0947	AZELAIC ACID	C9H16O4	1.76	0.006521
rp3315	22.42	287.1994	4-ANDROSTENE-3.17-DIONE	C19H26O2	1.69	0.006685
rp921	11.34	412.2092	PC 9:0	C17H34NO8P	1.65	0.015172
rp2469	18.86	235.1689	ASPERGILLUSENES A	C15H22O2	1.52	0.026676
rp2552	19.17	522.3546	LPC 18:1	C26H52NO7P	-1.57	0.026265
rp2548	19.15	480.3079	PE O-15:1_3:0	C23H46NO7P	-2.88	0.006521
rp2690	19.64	355.283	MONOLINOLEIN	C21H38O4	-3.14	0.013566
h60	11.07	191.019	CITRIC ACID	C6H8O7	-3.27	0.01465
h50	3.27	162.1118	CARNITINE	C7H15NO3	-3.44	0.039068
h197	15.94	421.0754	TREHALOSE-6-PHOSPHATE	C12H23O14P	-4.28	0.034181

## Discussion

The soil, the environment in which terrestrial plants anchor their roots, is a complex and densely populated ecosystem. It is home to a multitude of microbes that can have a neutral effect on the plant or engage in symbiotic interactions that can be beneficial or harmful (Morgan et al. 2005; Raaijmakers et al. 2008; Wang et al. 2024). In order to establish beneficial interactions in this highly populated environment, plants release specific chemical signals that are perceived by potential microbial partners, thereby inducing a response that can include the release of microbial signals, directional growth and metabolic changes that facilitate the establishment of symbiosis (Ortíz-Castro et al. 2009; Bonfante and Requena 2011; Genre et al. 2020).

The chemical signals, released by plants and microbes, that are necessary for the establishment of a symbiotic relationship have been identified and characterized in a limited number of interactions. These include the rhizobium/legume symbiosis and the arbuscular mycorrhiza (Limpens and Bisseling 2003; MacLean et al. 2017; Crosino and Genre 2022). Despite previous findings indicating that orchids may employ a genetic system of signal transduction similar to that identified in AM plants and legumes (Miura et al. 2018; Xing et al. 2020), the chemical signals involved in mutual recognition remain unknown, as do their influence on the cell organization and metabolism of the receiving partner. To the best of our knowledge, a single report suggests for SLs, the plant signal in AM (Kodama et al. 2022), a branch-inducing effect on *Armillaria mellea*, a fungus capable of forming ORM with the orchid *Gastrodia elata* (Yuan et al. 2018). However, *A. mellea* is an unusual ORM fungus, with a higher prevalence of reports associating it with root rot (Baumgartner et al. 2011).

In this study, we employed transcriptomics and metabolomics to examine the molecular alterations occurring in the ORM fungus *Tulasnella* sp. SV6 during the pre-symbiotic phase, preceding physical contact with the orchid roots. A transcriptomic comparison with the asymbiotic free-living mycelium, cultivated on the same medium in the absence of the orchid host, clearly showed substantial alterations in fungal gene expression in response to the plant’s presence. In particular, an increase in Differentially Expressed Genes (DEGs) involved in protein biosynthesis was observed, as evidenced by the enrichment of several Gene Ontology (GO) terms related to ribosome biogenesis and functioning, as well as the synthesis of several amino acids. Increased amino acid biosynthesis was also supported by metabolomic data, showing a higher content of some amino acids such as proline, pipecolinic acid (derived from lysine), phenylalanine, and some dipeptides. It is possible that some of the proteins induced in the ORM fungus during this pre-symbiotic stage may be involved in the symbiotic interaction. For example, one of the 20 most highly induced genes encodes a small secreted protein (transcript id: 246100, Log_2_FC: 7.16). Small secreted proteins (SSPs) are a common feature of the secretome of fungi from all lifestyles, representing between 40% and 60% of the secreted proteins in some cases (Feldman et al. 2020). SSPs may play a fundamental role in communication within a hyphal colony or with surrounding microbes in saprotrophic fungi (Plett and Plett 2022), but the complement of SSPs is typically increased in fungi interacting with plants, either as pathogens or as mutualistic symbionts (Kim et al. 2016). Novel functions for SSPs have been increasingly identified in plant-interacting fungi, where some strongly induced SSPs are used to manipulate the host defence response and to facilitate plant colonization (Kloppholz et al. 2011; Plett et al. 2011). Although the function of the *Tulasnella* sp. SV6 SSP remains to be established, its strong transcriptional upregulation in the presence of the orchid plantlet makes it an interesting candidate for further investigations of the pre-symbiotic events in ORM. Additionally, two genes encoding RlpA-like proteins were markedly upregulated in the PRESYMB sample (transcript id: 243232, Log_2_FC: 4.92 and transcript id: 240769, Log_2_FC: 1.64). In the pathogenic fungus *Rhizoctonia solani*, a related protein was identified as an effector capable of suppressing the plant immune response and was found to be highly induced during early-stage infection of sugar beet seedlings (Charova et al. 2020).

Carbohydrate Active enZymes (CAZymes) represent a significant portion of the fungal secretome. They are primarily involved in the saprotrophic degradation of complex polymers present in the substrate (Bonnin and Pelloux 2020; Gavande et al. 2023). However, fungal hyphae of mycorrhizal fungi interacting with plants must modulate the expression of these hydrolytic enzymes during interactions with the host (Veneault-Fourrey et al. 2014; Adamo et al. 2020; Chen et al. 2022). With regard to the repertoire of CAZymes regulated in the pre-symbiotic stage in *Tulasnella* sp. SV6, it is noteworthy that, among the most down-regulated genes, were those encoding pectinolytic enzymes, namely a pectin lyase (transcript id: 25833, Log_2_FC: -4.07) and a rhamnogalacturonase B (transcript id: 12253, Log_2_FC: -7.15). The reason for the strong downregulation of these pectinolytic enzymes in the pre-symbiotic stage is unknown, but since pectin is a major component of the plant cell wall, we cannot exclude that some recognition event may have occurred and led to a reduction of the fungal degradative potential towards plant cell wall components.

Metabolomic analyses revealed significant alterations in the lipid composition of the fungal mycelium, characterized by an increased abundance of glycerophospholipids (GP) such as phosphatidylcholines (PC) and lysophosphatidylcholines (LPC). Phosphatidylcholines are the principal structural membrane lipids, synthesized via diverse metabolic pathways in the Basidiomycetes (Kotlova et al. 2022). The accumulation of these compounds, in conjunction with the increased protein biosynthesis, may indicate stimulation of fungal growth in response to the host plant and the need for membrane biogenesis.

Our gene expression data corroborates the up-regulation of lipid biosynthesis, as DEGs coding for enzymes involved in phospholipid biosynthesis were up-regulated in the PRESYMB state (Table S4). For instance, a NAD(P)H-dependent glycerol-3-phosphate dehydrogenase, which generates a key precursor for phospholipid synthesis (transcript id: 3905, Log_2_FC: 4.13) was identified. Additionally, the data revealed the presence of a probable phospholipid-transporting ATPase IIB (transcript id: 109520, Log_2_FC: 2.47), as well as two phosphatidylserine decarboxylases (transcript id: 241123, Log_2_FC: 2.07; transcript id: 31445, Log_2_FC: 1.24).

The largest metabolomic differences observed in a previous study by Ghirardo et al. (2020) were also related to changes in the *Tulasnella* lipid content during asymbiotic and symbiotic growth. In that study, the ORM fungal mycelium was collected near orchid protocorms in which the mycorrhizal association was already established (i.e., a symbiotic condition), and compared with the free-living mycelium. By comparing our results with those of Ghirardo et al. (2020), PCs were found to increase their content in the *Tulasnella* mycelium in both symbiotic and pre-symbiotic conditions, as compared to the FLM. However, other lipids that increased their content in the fungal mycelium collected outside mycorrhizal protocorms (i.e., the symbiotic stage) were not found to increase in the mycelium collected in the pre-symbiotic stage. For example, a strong increase in sphingolipids (SP) and lysophosphatidylethanolamine (LPE) was detected in the symbiotic stage (Ghirardo et al. 2020), whereas we found no significant changes for these lipids in the pre-symbiotic ORM mycelium. Interestingly, the expression of a gene coding for a putative alkaline ceramidase, an enzyme that catalyzes the hydrolysis of ceramides into sphingosine, the precursor of sphingolipids, was downregulated in the pre-symbiotic stage (transcript id: 185418, Log_2_FC: -1.49). Further experiments are required to understand to what extent the different growth media and plant developmental stages (i.e., protocorms or adult plants) may influence the biosynthesis and accumulation of lipids in the ORM fungal mycelium, but the current data from this study and the previous one by Ghirardo et al. (2020) suggest that lipid composition may change in the external mycelium once the symbiosis with the plant is established.

Lipids are of significance not only as constituents of cellular membranes, but also as signalling molecules. Lysophosphatidylcholines play a significant role as signals and growth regulators in plants (Okazaki and Saito 2014), however their function in fungi remains less well-understood. A LPC(14:0-SN1) was found to be significantly accumulated in the PRESYMB samples (m.f. rp2307, Log_2_FC: 6.57). It is noteworthy that LPC has been observed to induce the expression of mycorrhiza-specific phosphate transporters in *Solanum tuberosum* roots colonized by AM fungi (Drissner et al. 2007; Vijayakumar et al. 2016). However, whether the LPC signal originates from the plant, the fungus, or a combination of both remains to be established.

Inositol phosphates are generated from phosphatidylinositol-4,5-bisphosphate (PIP2) through the action of phospholipase C and serve as recognized secondary messenger molecules in signal transduction and lipid signalling (Munnik and Testerink 2009). Two up-regulated DEGs coding for phospholipase C were identified in the *Tulasnella* sp. SV6 transcriptome, (transcript id: 31235, Log_2_FC: 2.06; transcript id: 21638, Log_2_FC: 1.92). The role of phospholipase C has been investigated in saprotrophic and pathogenic fungi belonging to diverse taxa, primarily through the disruption of PLC genes. The phenotype of these defective mutants indicate that phospholipases C in fungi are necessary for a number of cellular functions mainly related to fungal growth and hyphal development (Barman et al. 2018). A reduction in virulence was also typically observed in PLC mutants of pathogenic fungi. The up-regulation of the two *Tulasnella* sp. SV6 phospholipase C genes in the pre-symbiotic stage is therefore consistent with the general increase in biosynthetic activity, as revealed by both transcriptomics and metabolomics.

In conclusion, our findings demonstrate that the ORM fungus *Tulasnella* sp. SV6 exhibits alterations in its transcriptomic and metabolomic profiles when grown in the presence of the host plant *S. vomeracea*, before any physical contact with the host occurs. In comparison to the free-living mycelium, the fungus in the PRESYMB stage activates a biosynthetic apparatus involving protein and lipid biosynthesis. It is yet to be determined whether these changes are part of a specific response of the ORM fungus to symbiosis-related signalling molecules released by the host plant, or whether they reflect some modifications caused by the plant in the dual co-cultivation system, such as the release of nutrient-rich exudates or volatiles. It could be also speculated that the observed changes in amino acid metabolism may indicate not only an increased demands for fungal growth and signaling, but also that the fungus is preparing for nutrient transfer to the orchid. Previous work suggests that transfer of nitrogen to the host plant occurs in the form of amino acids (Dearnaley and Cameron 2017; Fochi et al. 2017a). Additionally, we cannot exclude that the presence of elevated CO_2_ concentrations may have influenced fungal growth and metabolic responses, a phenomenon observed in recent research by Chadwick and Lin (2024). However, during the daylight hours, photosynthesis by the plantlet likely mitigated CO_2_ enrichment, potentially limiting potential effects on the fungal response. Further investigations are required to address these questions.

## Electronic supplementary material

Below is the link to the electronic supplementary material.

**Supplementary Material 1: Figure S1.** *Tulasnella* sp. SV6 growing on 0.3% Oat Agar (a) and on M551 (b) media in Petri dishes

**Supplementary Material 2: Figure S2.** The different growth stages of *S. vomeracea* grown asymbiotically. a) Protocorms with leaf primordia 120 days after germination; b) *S. vomeracea* seedlings transferred in Falcon tubes; c) transfer of *S. vomeracea* seedlings to magenta jars under sterile conditions 20 days after growth in Falcon tubes; d) asymbiotic *S. vomeracea* plantlets in magenta jars.

**Supplementary Material 3: Figure S3.** The in vitro *Tulasnella*-*S.vomeracea* co-culture system; a) after co-inoculation (0 days post inoculum, dpi; and b) before sampling of the PRESYMB mycelium, 5 dpi.

**Supplementary Material 4: Tables S1–S5. Table S1.** List of pure internal standards used for data normalization. **Table S2.** Metaboscape 4.0 parameters used for processing LC-MS/MS data. **Table S3.** Read mapping metrics. **Table S4.** List of differentially expressed genes (DEGs) detected in PRESYMB condition compared to FLM. **Table S5.** List of mass features differentially accumulated detected in PRESYMB condition compared to FLM with p-adjusted value < 0.05.

## Data Availability

Reads from RNA-Seq were submitted to Sequence Read Archive (SRA) of NCBI under the BioProject ID PRJNA1194787.
