# Supplementary figures and images for: Omics approaches to investigate pre-symbiotic responses of the mycorrhizal fungus Tulasnella sp. SV6 to the orchid host Serapias vomeracea
Source: Mycorrhiza. 2025 Apr 2;35(2):26. doi: 10.1007/s00572-025-01188-6 (PMC11965168; doi:10.1007/s00572-025-01188-6)

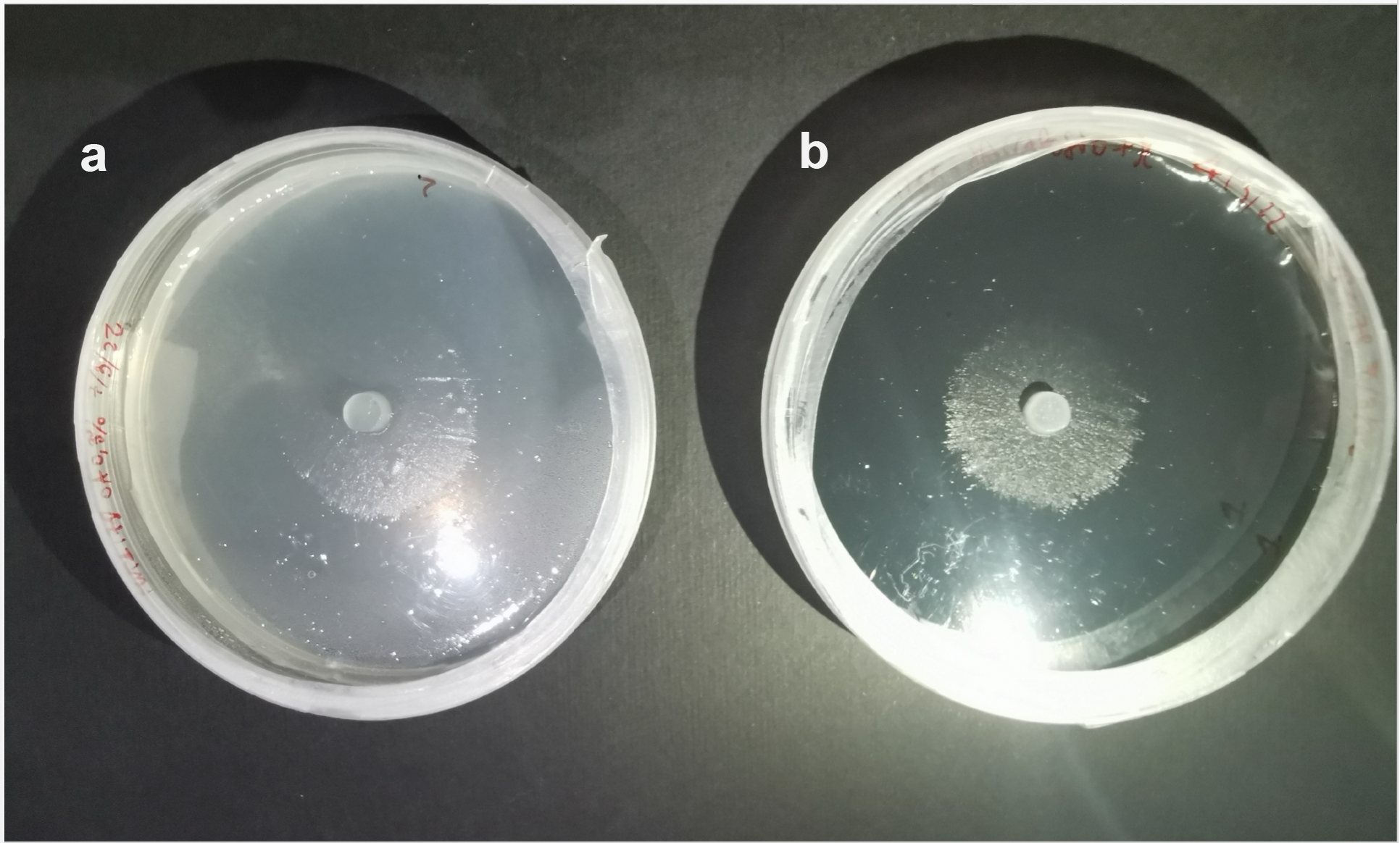

Supplement: Supplementary file 1 — Supplementary Material 1: Figure S1. Tulasnella sp. SV6 growing on 0.3% Oat Agar (a) and on M551 (b) media in Petri dishes [file 572_2025_1188_MOESM1_ESM.jpg]

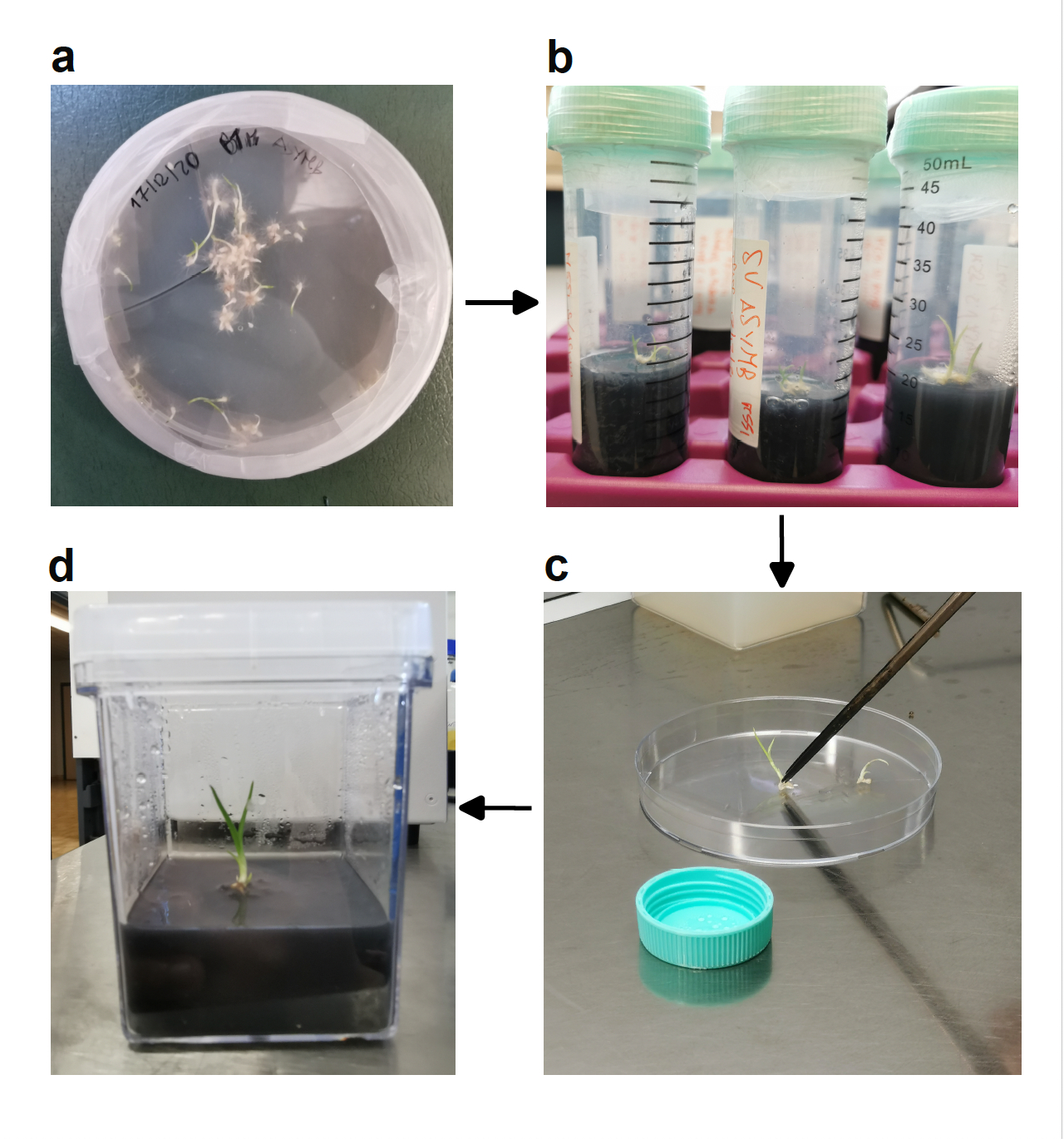

Supplement: Supplementary file 2 — Supplementary Material 2: Figure S2. The different growth stages of S. vomeracea grown asymbiotically. a) Protocorms with leaf primordia 120 days after germination; b) S. vomeracea seedlings transferred in Falcon tubes; c) transfer of S. vomeracea seedlings to magenta jars under sterile conditions 20 days after growth in Falcon tubes; d) asymbiotic S. vomeracea plantlets in magenta jars. [file 572_2025_1188_MOESM2_ESM.jpg]

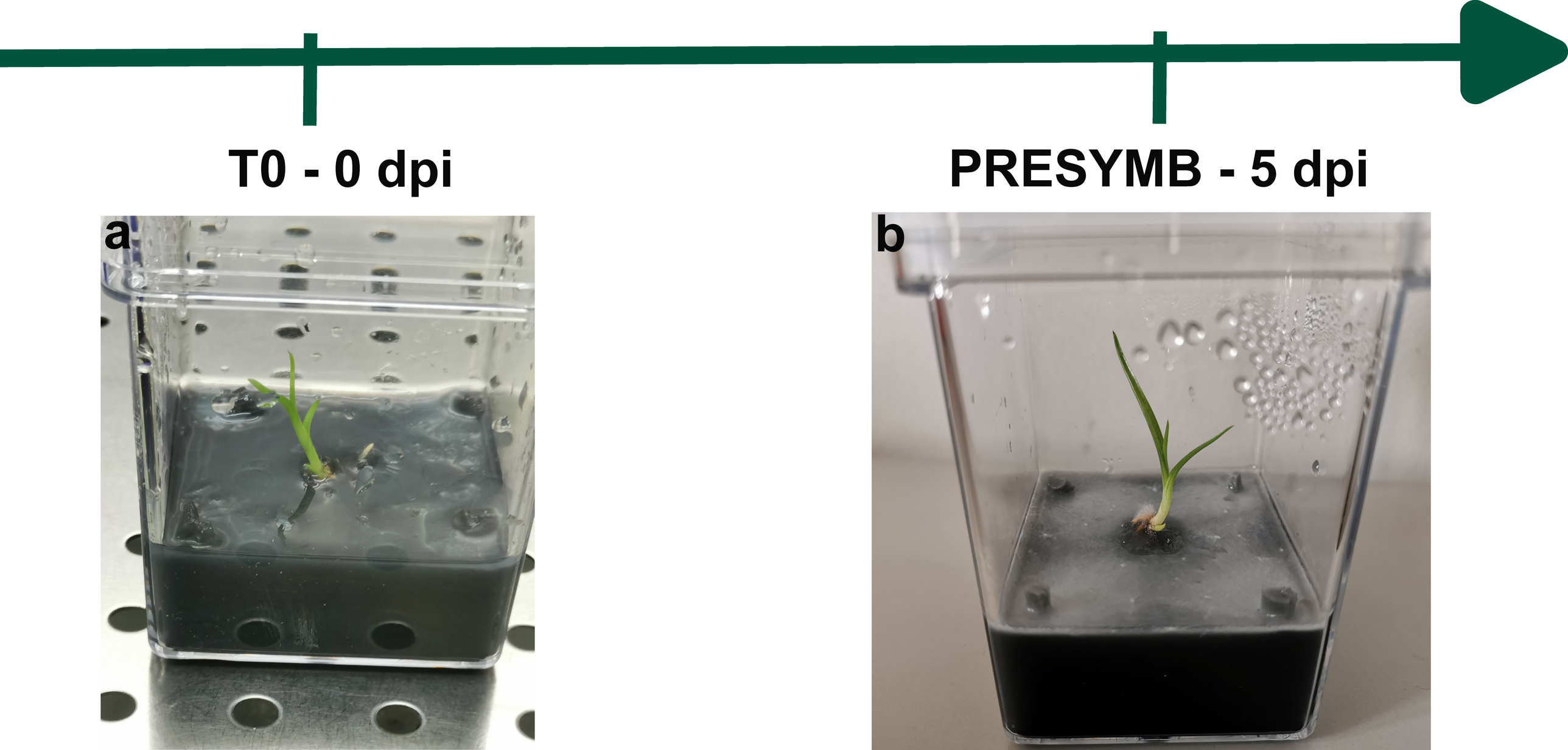

Supplement: Supplementary file 3 — Supplementary Material 3: Figure S3. The in vitro Tulasnella-S.vomeracea co-culture system; a) after co-inoculation (0 days post inoculum, dpi; and b) before sampling of the PRESYMB mycelium, 5 dpi. [file 572_2025_1188_MOESM3_ESM.jpg]
